# pH-Sensitive Polyampholyte Microgels of Poly(Acrylic Acid-*co*-Vinylamine) as Injectable Hydrogel for Controlled Drug Release

**DOI:** 10.3390/polym11020285

**Published:** 2019-02-08

**Authors:** Yanmin Chen, Peijian Sun

**Affiliations:** 1 College of Chemistry and Chemical Engineering, Zhengzhou Normal University, No.6 Yingcai Street, Zhengzhou 450044, China; yanminchen2011@163.com; 2 Zhengzhou Tobacco Research Institute of CNTC (China National Tobacco Corporation), No. 2 Fengyang Street, Zhengzhou 450001, China

**Keywords:** microgels, polyampholyte, poly(acrylic acid), polyvinylamine, injectable hydrogel, drug delivery

## Abstract

pH-sensitive polyampholyte microgels of poly(acrylic acid-*co*-vinylamine) (P(AA-*co*-VAm)) were developed as an injectable hydrogel for controlled drug release. The microgels of P(AA-*co*-VAm) were prepared via inverse suspension polymerization of acrylic acid and N-vinylformamide followed by hydrolysis of poly(*N*-vinylformamide) (PNVF) chains of the resultant microgels under basic condition. The pH-sensitivity of the P(AA-*co*-VAm) microgels in zeta potential and swelling ratio were investigated using a zeta potential analyzer and optical microscope. The results showed that both the zeta potential and the swelling ratio of the microgels were highly affected by the solution pH. By changing the pH of P(AA-*co*-VAm) microgel dispersion, the interparticle interaction and the swelling ratio of the microgels could be well adjusted and a colloidal hydrogel could be fabricated at moderate pH, showing a pH-triggered reversible fluid-gel transition. Using the polyampholyte P(AA-*co*-VAm) microgels as an injectable hydrogel drug release system, a sustained drug release could be achieved, indicating the great potentials of the pH-sensitive P(AA-*co*-VAm) microgels for controlled drug delivery.

## 1. Introduction

Hydrogels are insoluble polymer networks formed through the covalent or physical crosslinking of hydrophilic macromer precursors that can absorb a significant amount of water or biological fluids. Due to their excellent capability of retaining water and other biomimetic properties, hydrogels represent one of the most promising technology platforms for therapeutic intervention in a variety of diseases. They were widely explored in the domains of controlled drug delivery, tissue engineering, and regenerative medicine [1,2,3,4,5,6,7,8,9,10,11,12,13]. Among the varied hydrogel systems, the injectable hydrogels attracted much more attention due to their injectability, which is particularly useful for topical injection for a site-specific action [9,10,11,12,13]. These material systems are flowable aqueous solutions or dispersions before administration, but once injected, they rapidly turn into hydrogel under physiological conditions. The formation of hydrogels after injection brings forth some advantages: An injectable matrix can be implanted in the human body with minimal surgical wounds, and bioactive molecules or cells can be incorporated into the matrix simply by mixing before injection. After injection and gelation, these matrices could become drug delivery devices in pharmaceutics or cell-growing depots for tissue engineering. 

The injectable hydrogels can be formed by in situ chemical crosslinking or by the sol-gel or fluid-gel transition. In situ chemical crosslinking is a conventional approach to prepare a stable hydrogel [14,15,16,17,18,19]. For instance, the free radical chain growth polymerization of activated acrylates [16], click chemistry of alkynes and azides [17], and conjugate Michael addition of multifunctional thiol and activated -ene precursors [18,19] could result in stable hydrogels in situ. However, the suitability of some of these systems for in situ gelation was limited due to the incomplete conversion of reactive functional groups and high sol fraction. In addition, the use of metal catalysts, photo initiators, and/or ultraviolet light to initiate and propagate gelation also brought about biocompatibility concerns. In addition to chemically crosslinked hydrogels, in situ physically formed hydrogels may represent a much more promising injectable hydrogel technology platform for controlled drug delivery and tissue engineering. Physical gelation is free of any chemical reaction. Therefore, it could overcome the defects of some chemical gelatins that have biocompatibility problems of residue initiators or monomers. Some polymers in aqueous solution could undergo a reversible phase transition upon external stimuli or modest changes of environmental conditions like temperature, pH, redox condition, or ionic concentration, etc. For instance, the copolymers of poly(phenylene oxide)-poly(ethylene oxide) (PPO-PEO), poly(lactic-*co*-glycolic acid)-poly(ethylene oxide) (PLGA-PEO) and polycaprolactone-poly(ethylene oxide) (PCL-PEO) exited a sol-gel or fluid-gel transition upon a temperature stimulus [20,21,22]. They could turn into gel after injection into body and show great potentials in biomedical application. However, one great disadvantage of these systems is that they have to be heated up to a high temperature (e.g., 60 °C) during injection, which would cause necrosis, tissue scarring, and pain for the patient.

Polyelectrolytes are polymers whose repeating units bear electrolyte groups, such as carboxyl or amino groups. Generally, the weak polyelectrolyte has an average dissociation constant (p*K*_a_ or p*K*_b_) in the range of ~2 to ~10, which means that it would be partially dissociated at intermediate pH. Thus, the properties of weak polyelectrolytes, such as charge, can be modified by changing the solution pH. Among the varied polyelectrolytes, poly(acrylic acid) (PAA) is a common anion polyelectrolyte known for its pH-responsiveness, as it is composed polymeric backbones with pendant carboxyl groups. PAA is widely used in biomedical engineering, agriculture, and environmental protection [23]. Polyvinyl amine (PVAm) is a cationic polyelectrolyte that has the highest density of primary amine functional groups of any polymer. PVAm adsorbs spontaneously on most surfaces in aqueous solution, generating cationic interfaces. PVAm, therefore, provides a great potent tool for the modification of macroscopic and nanoparticle surfaces [24]. Using these polyelectrolytes, bulk complexes, such as hydrogels could be fabricated via the electrostatic attraction interactions between the oppositely-charges groups [25,26,27,28,29]. For instance, PVAm and poly(ethylene-*co*-maleic acid) (PEM) were used to coat poly(d,l-lactic-*co*-glycolic acid) (PLGA) microspheres with either positive or negative charges, respectively. The colloid self-assembled through electrostatic forces of the oppositely-charged PLGA microspheres resulted in a stable 3D network, showing great potential in controlled drug release and tissue engineering [28,29]. Polyampholyte microgel particles were crosslinked polyelectrolytes bearing both positively and negatively charged groups. They normally showed great pH-responsiveness: A minimum swelling ratio was normally observed at the isoelectric point (pI, the pH of zero net charge), while a much larger swelling ratio and high chain extension were shown at extreme pH values that far away the isoelectric point. These polyampholyte microgels found great application in drug delivery and metal-ion removal [30]. For example, poly(methacrylic acid)-poly[2-(dimethylamino)ethyl methacrylate](PAA-PDMAEMA) polyampholyte microgels were reported to have a pH-dependent swelling, uptake, and release behaviors, showing great potentials for controlled drug release [30,31].

In this report, we were interested in developing a novel injectable hydrogel system for controlled drug delivery. PAA and PVAm were chosen due to their polyanic and polycationic natures. Polyampholyte microgels of P(AA-*co*-VAm) were prepared via inverse suspension polymerization of acrylic acid and *N*-vinylformamide followed by hydrolysis under basic condition. The changes of swelling ratio and the zeta potential of the microgels upon the change of pH were investigated by optical microscope and zeta potential analyzer to test the pH-sensitivity. Finally, an injectable hydrogel system was assembled using the pH-sensitive polyampholyte microgels and the in vitro drug release behavior from the fabricated hydrogel was also investigated. 

## 2. Materials and Methods

### 2.1. Materials

*N*-Vinylformamide (NVF) and acrylic acid (AA) were purchased from Alfa Aesar (Tianjin, China). NVF was distilled under vacuum and stored at −20 °C, while AA was passed through an alumina column before use. 4,4’-azobis(4-cyanopentanoic acid) (V501), *N*,*N*’-methylenebisacrylamide (MBA) and Span 60, from Sigma-Aldrich (Shanghai, China), were used as supplied. Other reagents were commercially available chemicals and used as received.

### 2.2. Preparation of P(AA-co-NVF) and P(AA-co-VAm) Microgels

The P(AA-*co*-NVF) microgels were prepared by inverse suspension polymerization of AA and NVF with MBA as crosslinker [32]. Typically, the mixture of methanol-water (*v*/*v* = 1/4, 25 mL), monomers (AA, 25 mmol; NVF, 25 mmol), cross-linker (MBA, 1.5 mmol), and initiator (V501, 0.5 mmol) was added to a pre-purged oil phase containing 250 mL of heptane, 7.5 g Span 60 under stirred at 400 rpm. The polymerization was conducted at 65 °C for 3 h at a stirring speed of 400 rpm. The resultant microgels were separated by centrifuge at 4000 rpm for 10 min, washed with distilled water, acetone and ethanol for several times. The P(AA-*co*-NVF) microgels were obtained as white powder by drying under vacuum for 24 h at 40 °C.

The P(AA-*co*-VAm) microgels were prepared by basic hydrolysis of P(AA-*co*-NVF) microgels [33]. The P(AA-*co*-NVF) microgels (1.0 g) were dispersed in 100 mL of 2M NaOH solution and kept at 80 °C for 5 h. The resultant P(AA-*co*-VAm) microgels were separated by centrifuge at 4000 rpm for 10 min, washed thoroughly with distilled water, neutralized, and purified by dialysis for a week against regularly replaced pure water. The P(AA-*co*-VAm) microgels were recovered by lyophilization.

### 2.3. Characterization of Microgels

FTIR spectra were recorded in KBr disks using a Perkin-Elmer Spectrum One instrument (Perkin-Elmer, Waltham, MA, USA). The average number of scans per spectrum was 64 and the spectral resolution was 4 cm^−1^. The surface structure of the microgels was characterized with an S-4700 (Hitachi, Tokyo, Japan) scanning electron microscope (SEM). The samples were mounted directly onto the SEM sample holder using double-sided sticking tape and were sputter-coated with gold in vacuum prior to measurements. Zeta potential of the microgels was measured by laser Doppler electrophoresis using a Zetasizer Nano ZS instrument (Malvern instrument, Malvern, UK) at 25 °C. For samples prepared, the microgels were dispersed into phosphate buffer (PB, 50 mM) solutions with different pH at a microgel concentration of 0.3 mg/mL for 24 h prior to measurements, the large particles were removed by sedimentation, and the upper solution was injected into a disposable cuvette for the measurement of zeta potential. Optical morphologies of the swollen microgels were observed using a BX71 optical microscope (Olympus, Hachioji, Japan) and the diameters of the microgels were statistic from the optical images. The swelling ratios (*q*) of the microgels were estimated using Equation (1)

(1) $$q={\left(\frac{d}{d_{c}}\right)}^{3}$$

where *d_c_* is the collapsed particle diameter that determined by SEM, *d* is the swollen particle diameter determined by optical microscope images. 

### 2.4. Fluid-Gel Transition

The fluid-gel transition behavior of P(AA-*co*-VAm) microgels in aqueous dispersion was determined using the test tube inverting method. The microgels were dispersed in water at pH 3 with a desired concentration. Then, the pH of the solution was increased by adding a small amount of 4M NaOH. After kept for 10 min at the pH, the fluid-gel transition was determined by angling the vial horizontally. A gel was determined when no significant flow was observed 30 s after the vial was inverted.

### 2.5. In Vitro Drug Release from the Hydrogels

The release profiles of drug from hydrogels were studied at 37 °C in phosphate-buffered solution with different pH. Briefly, 1.0 mL of hydrogels of 3.5% and/or 2.5% P(AA-*co*-VAm) were prepared by adjusted the pH to the desired pH value in a tube. Doxorubicin (DOX) were incorporated into the hydrogels during the fluid-gel transition with a payload of 400 µg. Then, 2.0 mL of phosphate-buffered solution was added to the upper of the hydrogel. At selected time intervals, 1.0 mL of the upper solution was removed for UV–vis analysis and replaced with fresh phosphate-buffered solution. DOX concentration was calculated based on the absorbance intensity at 480 nm. In the assessment of drug-release behavior, the cumulated amount of released drug was calculated, and the percentages of drug released were plotted against time. 

## 3. Results and Discussions

### 3.1. Preparation and Characterization of the Microgels

**Scheme 1:**
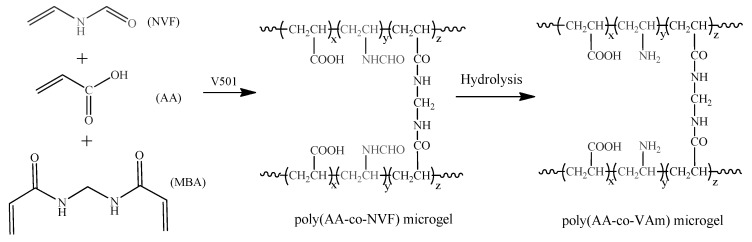
Preparation pathway of P(AA-*co*-NVF) and P(AA-*co*-VAm) microgels.

Scheme 1 shows the preparation pathway of the P(AA-*co*-NVF) and P(AA-*co*-VAm) microgels. The P(AA-*co*-NVF) microgels were prepared by inverse suspension copolymerization of AA and NVF with MBA as crosslinker. Then, hydrolysis of the P(AA-*co*-NVF) microgels under basic condition gave the corresponding P(AA-*co*-VAm) microgels.

**Figure 1:**
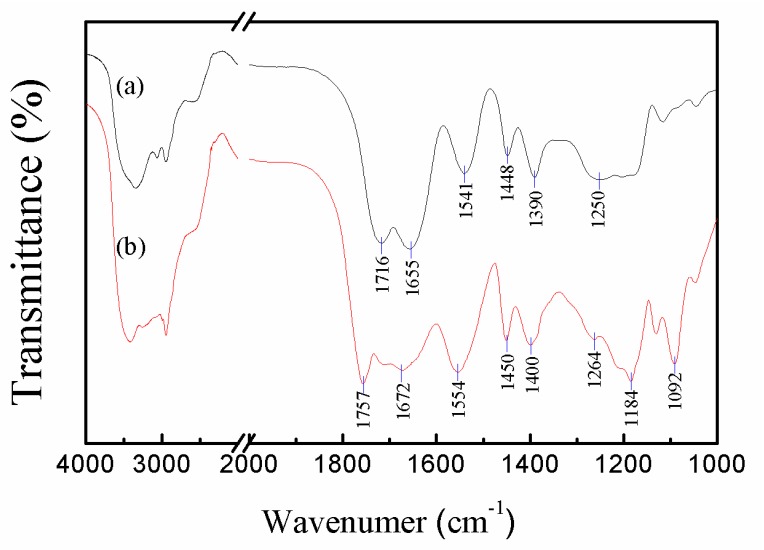
FTIR spectra of the (**a**) P(AA-*co*-NVF) and (**b**) P(AA-*co*-VAm) microgels.

Figure 1 showed the typical FTIR spectra of the P(AA-*co*-NVF) and P(AA-*co*-VAm) copolymer microgels with characteristic peaks shown in the spectra. As for the P(AA-*co*-NVF) microgel, the amide and carboxylic acid adsorption peaks, which are specific to NVF and AA components, respectively, can be identified in the FTIR spectrum. The amide peak of the NVF group appeared at 1655 cm^−1^ for amide I (–CO–NH–, C=O stretching) and at 1541 cm^−1^ for amide II (–CO–NH–, N–H bending). Characteristic of C=O and C–O stretching bands of the carboxylic acid group of AA appeared at 1716 and 1250 cm^−1^, respectively. The FTIR result indicated successful polymerization of NVF and AA as expected. Basic hydrolysis of P(AA-*co*-NVF) microgels was utilized to prepare P(AA-*co*-VAm) microgels. After hydrolysis, in the FTIR spectrum, characteristic of C=O and C–O stretching bands of the carboxylic acid group of AA shifted to 1757 and 1264 cm^−1^, these blue shifts were due to the replace of hydrogen bond interaction by electrostatic interaction in the copolymer network. The character 1655 and 1541 cm^−1^ absorption for amide of NVF group disappeared and a new group of absorption peak belonged PVAm at 1672 cm^−1^ (N–H deformation vibration), 1554 cm^−1^ (C–N stretching) appeared, indicating the success preparation of the P(AA-*co*-VAm) microgels. 

**Figure 2:**
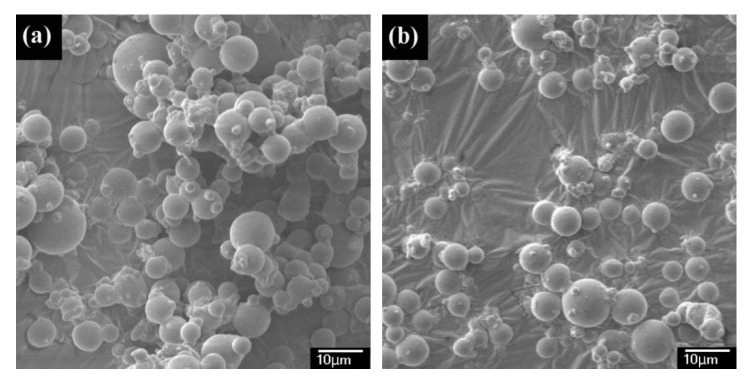
SEM images of (**a**) P(AA-*co*-NVF) and (**b**) P(AA-*co*-VAm) microgels.

Figure 2 shows the typical SEM images of the P(AA-*co*-NVF) and P(AA-*co*-VAm) microgels. Both the P(AA-*co*-NVF) and P(AA-*co*-VAm) microgels have regularly spherical shapes with relatively smooth surfaces, and the microgel particles have average sizes of 4.7 μm for P(AA-*co*-NVF) and 3.8 μm for P(AA-*co*-VAm) microgels, respectively.

### 3.2. pH-Sensitivity of the Microgels

The microgels of P(AA-*co*-NVF) and P(AA-*co*-VAm) bear electrolyte groups, such as carboxyl and/or amino groups, which would be partially dissociated at intermediate pH. Thus, the properties of these polyelectrolytes microgels, such as charge and swelling behaviors, would be greatly affected by the solution pH. 

**Figure 3:**
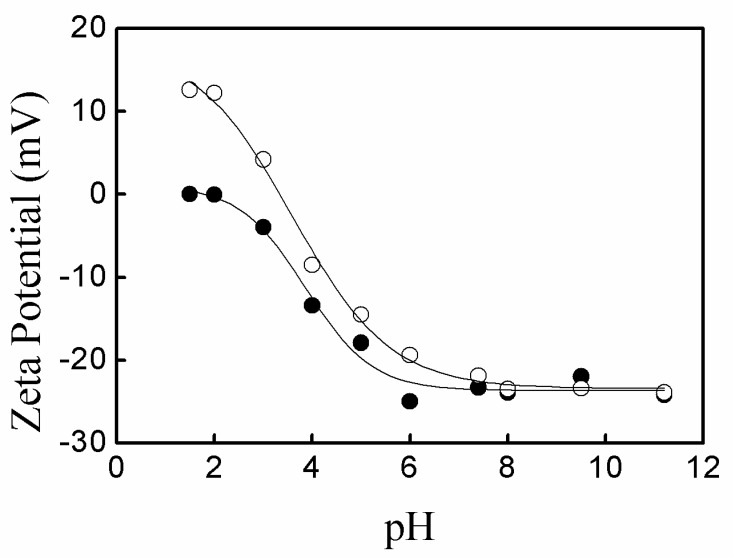
Variation of the zeta potential of P(AA-*co*-NVF) (solid) and P(AA-*co*-VAm) (hollow) microgel as a function of pH.

Figure 3 shows the zeta potential of P(AA-*co*-NVF) and P(AA-*co*-VAm) microgels at various pH. The P(AA-*co*-NVF) microgel have a zeta potential of about −25 mV at pH above 6.0 due to the nearly complete ionization of carboxyl groups in PAA with a p*K*_a_ of ~4.5. The zeta potential of P(AA-*co*-NVF) increased sharply with pH decrease from 6.0 to 2.0 and reached near 0 mV at pH 1.5~2.0 due to the near complete protonation of carboxyl groups in PAA. In contrast to P(AA-*co*-NVF) microgels, an isoelectric point was observed at pH 3.5 for P(AA-*co*-VAm) microgels, where the net charge is zero. Below the isoelectric point of pH 3.5, the zeta potential increased greatly to about 12 mV upon the pH decrease from 3.5 to 1.5. Above the isoelectric point of pH 3.5, a negative zeta potential was observed and the zeta potential decreased largely from 0 mV to about −25mV, while the pH increased from 3.5 to 8.0, and a slight decrease of zeta potential was observed from pH 8.0 to pH 11.5. The change of zeta potential is due to the ionization balance of the carboxyl groups in PAA and primary amino groups in PVAm. PAA has an average dissociation constant of pKa ≈ 4.5 while the dissociation constant of the protonated PVAm (PVAm·H^+^) is of pKa ≈ 8.0 [24]. At pH 3.5, the carboxyl groups in PAA were partially ionized, creating a negative charge. Meanwhile, the primary amino groups in PVAm were partially protonated, providing a positive charge. The positive and negative charges wiped one another out and gave a zero net charge. Below the isoelectric point, the amount of negatively charged ionized carboxyl groups decreased, and the amount of positively charged protonated amino groups increased with the decrease of pH, which resulted in a net positive zeta potential and the increase of zeta potential for the P(AA-*co*-VAm) microgels upon the pH decreased from 3.5 to 1.5. Above the isoelectric point, the amount of negatively charged ionized carboxyl groups increased and the amount of positively charged protonated amino groups decreased upon the pH increase from 3.5 to 8.0, resulting in that the zeta potential decreased from 0 mV to about −25 mV with pH increasing from 3.5 to 8.0. When pH was above 8.0, almost all the carboxyl groups were ionized, while the small fraction of the protonated primary amino groups decreased upon the pH increase from 8.0 to 11.5. As a result, the zeta potential decreased slightly with pH increase from 8.0 to 11.5.

**Figure 4:**
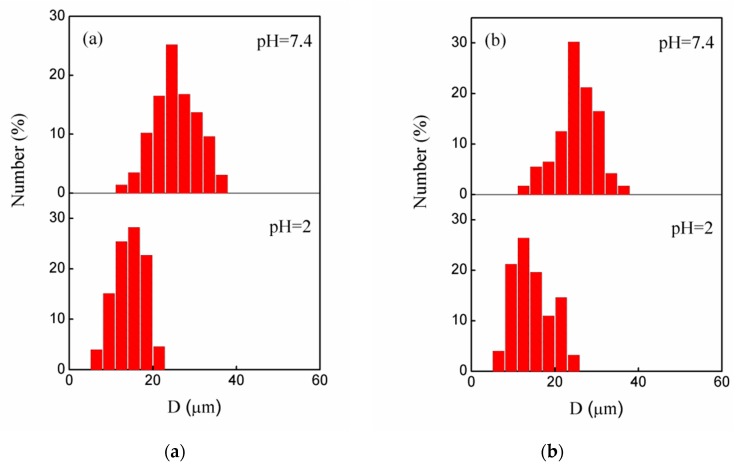
Size distribution of (**a**) P(AA-*co*-NVF) and (**b**) P(AA-*co*-VAm) microgels at different pH.

**Figure 5:**
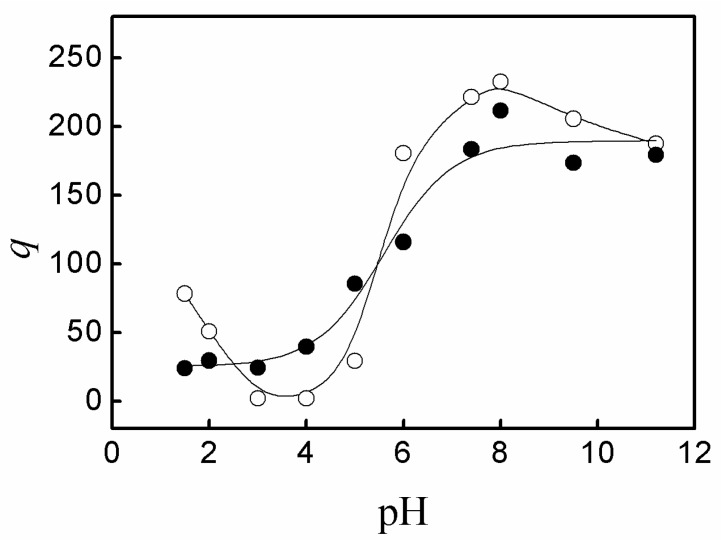
Variation of the swelling ratio (*q*) of P(AA-*co*-NVF) (solid) and P(AA-*co*-VAm) (hollow) microgel as a function of pH.

Figure 4 showed the size distribution of the swollen P(AA-*co*-NVF) and P(AA-*co*-VAm) microgels in different pH solution, as statistic from the optical images. The P(AA-*co*-NVF) microgels have an average swollen diameter (*d*) of 14.5 μm at pH 2.0 and 26.7 μm at pH 7.4, while the P(AA-*co*-VAm) microgels have an average swollen diameter of 14.8 μm at pH 2 and 24.2 μm at pH 7.4. The variation of the swelling ratio (*q*) as a function of pH is shown in Figure 5. The P(AA-*co*-NVF) microgels were swollen most at pH above 7.4 (*q* = 175), exhibited a significant decrease in swelling ratio at pH = 6, and reached a constant of about 25 at pH blow 4. As for P(AA-*co*-VAm) microgels, the highest swelling ratio was observed at pH 7~8, and a large decrease in the swelling ratio was observed at pH below 6 and pH above 9. The swelling ratio of P(AA-*co*-VAm) reach a minimum at pH between 3 and 4, nearing the isoelectric point. Below the isoelectric point, the swelling ratio increased significantly with a pH decrease from pH 3 to pH 1.5. The pH-dependent of swelling behaviors were due to the different polyelectrolytes nature. P(AA-*co*-NVF) microgel is a polyanionic polymer network. The carboxyl in PAA is nearly complete ionization at pH above 6, resulting a higher osmotic pressure and consequently a higher swelling ratio. The swelling ratio decreased upon the pH decrease, which resulted from the protonation of carboxyl in PAA. P(AA-*co*-VAm) microgels are a polyampholyte polymer network bearing both carboxyl and amino groups. The P(AA-*co*-VAm) microgel reaches its isoelectric point at a certain pH (e.g., pH = 3.5), where the osmotic pressure reaches a minimum. Thus, the swelling ratio of P(AA-*co*-VAm) microgels also reached a minimum at the isoelectric point. When the pH decreased from 3.5 to 1.0, the protonation of amino groups in PVAm resulted in a higher osmotic pressure, and thus, a higher swelling ratio; Similarly, with the pH increased from 3.5 to 8.0, the ionization of carboxyl groups in PAA also resulted a higher osmotic pressure and a higher swelling ratio.

### 3.3. Fluid-Gel Transition of P(AA-co-VAm) Microgels

**Figure 6:**
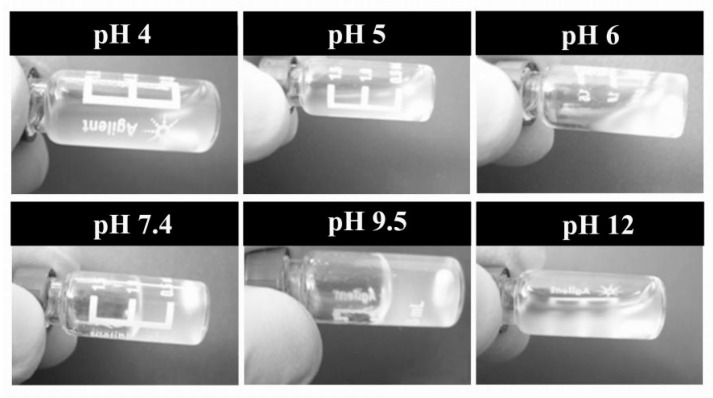
Digital images of P(AA-*co*-VAm) microgels at varied pH. The concentration of microgels is 2.5 wt %.

Figure 6 shows the digital images of P(AA-*co*-VAm) microgels dispersion with a 2.5% polymer concentration at varied pH. The dispersion of P(AA-*co*-VAm) microgels was flowable at pH below 6.0. When the pH was increased to 7.4 and/or 9.5, the dispersion turned from fluid into a gel state. With a much higher value of pH 12, the formed hydrogel could turn into fluid again. Interestingly, the destroyed hydrogels (e.g., at pH 12) could be reformed into a gel state by changing the pH to the moderate pH region (such as pH 7.4 or 9.5), which meant that the fluid-gel transition is reversible. However, no fluid-gel transition was observed for the P(AA-*co*-NVF) microgels solution. The fluid-gel transition of P(AA-*co*-VAm) microgels dispersion resulted from the pH-dependent electrostatic interactions and swelling behavior of the polyampholyte microgels.

As discussed above, the microgels of P(AA-*co*-VAm) bared both carboxyl and amino groups inside the polymeric network. PAA has an average dissociation constant of pKa ≈ 4.5, while the dissociation constant of the protonated PVAm (PVAm·H^+^) is of pKa ≈ 8.0 [24]. The existence forms of both carboxyl (e.g., –COOH or –COO^−^) and amino groups (e.g., –NH_3_^+^ or –NH_2_) inside the polymeric network are highly affected by pH, which resulted in that the electrostatic interactions between the microgels are highly affected by the solution pH. At a much lower pH (e.g., pH = 2), both the carboxyl groups and amino groups were almost entirely protonated as –COOH and –NH_3_^+^, respectively. As a result, the microgels had a high positive charge (e.g., 12 mV) in the polymeric network, resulting in the electrostatic repulsion between the microgels. In addition, the microgels were not swollen in large quantities, as compared to the high pH range (e.g., pH = 8). These phenomena resulted in that the microgel dispersion could flow easily at a rather low pH. At pH = 4, nearing the isoelectric point, the much lower swelling ratio of the microgels also resulted in the flowability of the microgel dispersion. However, at moderate pH (e.g., pH 7.4), the carboxyl groups were ionized into –COO^−^ and the amino groups were partially protonated into –NH_3_^+^, which resulted into that the microgels had the negative and positive charges in the polymeric network. As a result, the microgels could interact with each other by the high electrostatic attraction and fabricate in a hydrogel. In addition to the high electrostatic interactions, the much higher swelling ratio of the microgels also has a positive contribution to the forming of hydrogel at moderate pH. As for a much higher pH (e.g., pH 12), the carboxyl groups were ionized entirely as –COO^−^ and the amino groups were almost neutralized as –NH_2_, showing high negative charges in the polymeric network, which resulted in the electrostatic repulsion between the microgels. In addition, the size of the swollen microgels decreased a bit due to the salt effect. As a result, the P(AA-*co*-VAm) microgel dispersion turn into fluid at much higher pH (e.g., pH 12).

**Figure 7:**
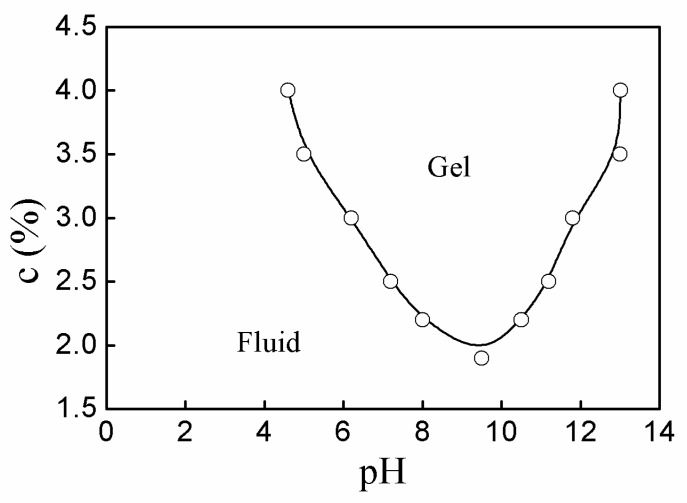
The phase diagram of the aqueous dispersion of P(AA-*co*-VAm) microgels.

Figure 7 shows the phase diagram of fluid-gel transition of the P(AA-*co*-VAm) microgels aqueous dispersion. The fluid-gel transition was greatly affected by the pH and the polymer concentration. For example, the P(AA-*co*-VAm) microgel dispersion with a 2.2% concentration was a flowable fluid at pH < 8.0, and it could turn into the hydrogel at pH > 8.0, which could be destroyed into a flowable dispersion at pH > 10.5. Notably, the P(AA-*co*-VAm) microgel dispersion had a narrow pH window to form hydrogels at lower polymer concentration, while the dispersion with a high polymer concentration could turn into hydrogel at a relatively wide pH region. For instance, the pH region for the P(AA-*co*-VAm) microgel dispersion of 2.2% concentration to form hydrogel was about pH 8.0–10.5. However, the pH region for a 3.0% P(AA-*co*-VAm) microgel dispersion to form hydrogel is of 6.2–11.8, which was much wider than that of a lower polymer concentration. With a higher polymer concentration of 3.5% or 4.0%, the dispersion could form hydrogel at a relatively wide pH region of pH > 5.0 or pH > 4.6, and the hydrogels were stable even at pH as high as 13. The wide pH windows for aqueous dispersion with a higher microgel concentration to form hydrogels is related to much more charged microgel particles in the dispersion and to the high adsorption of aqueous solution the microgel at a higher pH. With a higher polymer concentration, there are much more charged microgel particles in the dispersion, and the charged particles could electrostatically interact with each other to form an unflowable gel in spite of the inadequate swelling of the microgels in lower pH region, which resulted in the decrease of the lower boundary of the fluid-gel transition for a higher microgel concentration. On the other hand, with a higher polymer concentration, almost all the aqueous solution could be adsorbed by the microgels at high pH region. The tremendously swollen microgels interacted with each other via electrostatic interaction and/or adhesion and mutual friction interaction, which resulted in that the dispersion could keep a gel state at a high pH region (e.g., as high as pH 13) and an increase of the upper boundary of the fluid-gel transition for a higher microgel concentration.

### 3.4. In Vitro Drug Release

**Figure 8:**
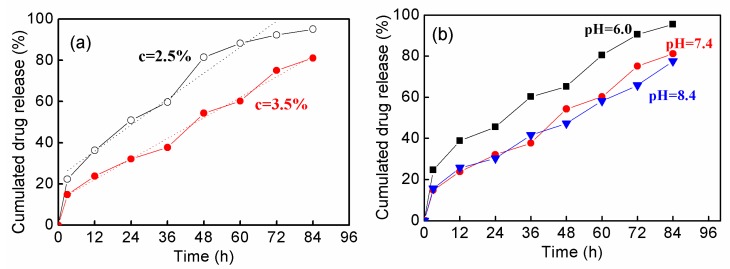
The drug release profiles from the hydrogels fabricated by P(AA-*co*-VAm) microgels. (**a**) Drug released from P(AA-*co*-VAm) microgels with 2.5% and 3.5% polymer concentration at pH 7.4. The dotted lines were the linear fitting results. (**b**) Drug released from P(AA-*co*-VAm) microgels with 3.5% polymer concentration at different pH levels.

Figure 8 shows the in vitro release of DOX from hydrogels fabricated by P(AA-*co*-VAm) microgels. The drug release profiles were dependent on both the microgel concentration and the pH. 

As seen in Figure 8a, DOX release much faster from the hydrogel with a lower microgel content (e.g., 2.5%) than that of a higher microgel content (e.g., 3.5%) at pH 7.4. For example, 32% of DOX released from the hydrogel assembled by 3.5% microgels in 24 h, while much more DOX (51%) released from the hydrogel with a 2.5% microgel content. This is probably due to the fact that the hydrogel with lower microgel content is easily eroded due to its lower stability, which resulted from the fact that the release was performed at the pH nearing the hydrogel forming pH boundary. Thus, a faster release could be achieved form the hydrogel with a lower microgel content. It was further observed that DOX was released from the hydrogels in a near-zero ordered manner up to 80% release for the hydrogels with either a 2.5% or 3.5% polymer content. The sustained near-zero ordered drug release profile should be controlled by the erosion of hydrogel. The erosion of hydrogel was taken at the top hydrogel surface with a constant area of release, resulting in a near-zero ordered drug release.

The drug release from hydrogels fabricated by 3.5% P(AA-*co*-VAm) microgels was investigated at different pH levels. As shown in Figure 8b, the drug released slower at pH 7.4 and pH 8.4 than at pH 6.0. The pH-dependent drug release should resulted from the difference in the stability of the forming hydrogels with different pH levels. The formed hydrogel at pH 6.0 had a lower stability and the erosion of the hydrogel was easier due to the pH value is near the lower boundary of the fluid-gel transition. At a higher pH of 7.4 or 8.4, the stability of the formed hydrogels is higher and consequently a long-term drug release could be achieved at pH of 7.4 or 8.4.

Therefore, in combination of the favorable properties including injectability, pH-triggered fluid-gel transition, and sustained drug release, the pH-sensitive polyampholyte microgels of P(AA-*co*-VAm) showed great potentials for injectable drug delivery. 

## 4. Conclusions

pH-sensitive polyampholyte microgels of P(AA-*co*-VAm) were developed as injectable hydrogel for controlled drug release. The P(AA-*co*-VAm) microgels were prepared via inverse suspension polymerization of AA and NVF, followed by hydrolysis under basic condition. Both the swelling ratio and the zeta potential of the P(AA-*co*-VAm) microgels were highly affected by the solution pH, showing pH-sensitivity. By changing the pH, the P(AA-*co*-VAm) microgel dispersion showed a pH-triggered fluid-gel transition, and a colloidal gel could be assembled at moderate pH values. Using the polyampholyte P(AA-*co*-VAm) microgels as injectable hydrogel drug release system, a sustained drug release could be achieved, showing the great potential of these pH-sensitive polyampholyte microgels for controlled drug delivery.
